# The Emerging Plasticizer Alternative DINCH and Its Metabolite MINCH Induce Oxidative Stress and Enhance Inflammatory Responses in Human THP-1 Macrophages

**DOI:** 10.3390/cells10092367

**Published:** 2021-09-09

**Authors:** Alexandra Schaffert, Josi Arnold, Isabel Karkossa, Matthias Blüher, Martin von Bergen, Kristin Schubert

**Affiliations:** 1 Department of Molecular Systems Biology, Helmholtz-Centre for Environmental Research (UFZ), 04318 Leipzig, Germany; alexandra.schaffert@ufz.de (A.S.); josi.arnold@ufz.de (J.A.); isabel.karkossa@ufz.de (I.K.); martin.vonbergen@ufz.de (M.v.B.); 2 Helmholtz Institute for Metabolic, Obesity and Vascular Research (HI-MAG), 04318 Leipzig, Germany; matthias.blueher@uniklinik-leipzig.de; 3 Department of Endocrinology, Nephrology Rheumatology, University Hospital Leipzig Medical Research Center, 04318 Leipzig, Germany; 4 Institute of Biochemistry, Leipzig University, 04103 Leipzig, Germany

**Keywords:** DINCH, plasticizer, macrophage, proteomics, immunotoxicity

## Abstract

The use of the plasticizer bis(2-ethylhexyl)phthalate (DEHP) and other plasticizers in the manufacture of plastic products has been restricted due to adverse health outcomes such as obesity, metabolic syndrome, and asthma, for which inflammation has been described to be a driving factor. The emerging alternative plasticizer 1,2-cyclohexanedioic acid diisononyl ester (DINCH) still lacks information regarding its potential effects on the immune system. Here, we investigated the effects of DINCH and its naturally occurring metabolite monoisononylcyclohexane-1,2-dicarboxylic acid ester (MINCH) on the innate immune response. Human THP-1 macrophages were exposed to 10 nM–10 μM DINCH or MINCH for 4 h, 16 h, and 24 h. To decipher the underlying mechanism of action, we applied an untargeted proteomic approach that revealed xenobiotic-induced activation of immune-related pathways such as the nuclear factor κB (NF-κB) signaling pathway. Key drivers were associated with oxidative stress, mitochondrial dysfunction, DNA damage repair, apoptosis, and autophagy. We verified increased reactive oxygen species (ROS) leading to cellular damage, NF-κB activation, and subsequent TNF and IL-1β release, even at low nM concentrations. Taken together, DINCH and MINCH induced cellular stress and pro-inflammatory effects in macrophages, which may lead to adverse health effects.

## 1. Introduction

As part of the innate immune system, macrophages are found in most organs and tissues of the body [1]. They are critical for modulating immune responses and release chemokines and cytokines upon activation [1]. Activation can be facilitated by environmental hazards, which can be microorganisms as well as xenobiotics [2]. One group of xenobiotics, the plasticizers, has previously been described to modulate immune responses and enhance susceptibility to pathogens or promote chronic inflammation in tissues, which is associated with concomitant diseases such as cardiovascular diseases, type 2 diabetes, asthma, and allergies [2,3]. 

Plasticizers are non-covalently bound components of plastic products that are used in every area of life, including food packaging, textiles, building materials, children’s utensils, and medical items [4]. Over time, plasticizers can leach from these plastic products and expose humans through air, food, or skin contact [5]. Several plasticizers are considered harmful to human health by acting as endocrine disruptors (EDCs), interfering directly with receptors or signaling molecules, and modulating hormone production or transport [6]. The immune system is inherently connected to the endocrine system, i.e., immune cells can not only provide immunoregulatory compounds as well as a variety of hormones [7], but can also be influenced by endogenous hormones as well as xenobiotics [3,8]. Several studies have reported that plasticizers alter the innate immune response by affecting phagocytosis capacity and cytokine secretion, although the underlying mechanisms remained unclear [9,10,11]. In particular, bis(2-ethylhexyl) phthalate (DEHP), the most widely used plasticizer, has been shown to increase the secretion of proinflammatory chemokines and cytokines ex vivo in rat macrophages and in vitro in human macrophages, mediated by nuclear factor-κB (NF-κB) activation [12,13].

After the European Union restricted the use of DEHP and other known endocrine disrupting phthalates, they have been increasingly replaced by alternative compounds, one of which is 1,2-cyclohexanedicarboxylic acid diisononyl ester (DINCH) [14]. DINCH is used in food packaging, children’s toys, medical materials such as infusion tubes and blood bags, printing inks, paints, and textile coatings [15]. Global production of DINCH increased from an initial 25,000 metric tons per year in 2002 to 200,000 metric tons per year in 2014, resulting in a rapid increase in the detection of DINCH transformation products in urine samples to nearly 100% of samples [16]. This steady increase in exposure creates the need to investigate the potential adverse effects of DINCH in humans. To date, there are no restrictions on DINCH consumption or production [15].

Recent studies conclude that DINCH has more significant effects on an organism than previously assumed [14,17]. According to Campioli et al. [17], DINCH impairs metabolic pathways of primary liver cells in rats at a dose of 1 mg/kg body weight/day via activation of the peroxisome proliferation-activating nuclear receptor α (PPARα), similar to DEHP. DINCH also directly affects hormone-producing Leydig cells in the testes of rats after prolonged exposure, causing a decrease in blood testosterone levels [14]. However, adverse effects of DINCH on the immune response have not yet been investigated. Since DINCH has already been shown to have comparable endocrine effects to other xenobiotics and similarity in chemical structure to common plasticizers is evident, it is reasonable to assume that DINCH may also have adverse effects on the immune response. This demonstrates the need to look more closely at the effects of DINCH with respect to the immune system. In addition, it is vital to include biotransformation products of a plasticizer in the investigation, since many phthalates have been shown to cause more potent adverse effects than their parent compounds [18]. Therefore, we aimed to investigate the adverse effects of DINCH and cyclohexane-1,2-dicarboxylic acid monoisononyl ester (MINCH), which is a major biotransformation product of DINCH [19], on the immune response of the human macrophage cell line THP-1 and the underlying mechanisms.

Since DINCH concentrations in human blood have not yet been published, we considered blood levels of common plasticizers corresponding to a low-to-moderate nM level to study DINCH [20]. An initial in vitro assessment contributes to the risk assessment of DINCH. To make the results from this initial assessment as comparable as possible, a model system should be used for this purpose, which is widely applicable and readily available. Both of these points are true for the widely used and well-established THP-1 cells, rendering them a useful model system for initial studies of the adverse effects of xenobiotics in human cells and making them more suitable for an initial risk assessment than primary cells. A variety of studies have found immunomodulatory effects of plasticizers using THP-1 cells [10,12,21]. Therefore, performing initial assessments with the same cell line ensures comparability of common, well-studied plasticizers with DINCH, which is useful for assessing whether it is a safer alternative. Investigating pro-inflammatory M1 macrophages is especially critical, since they are involved in chronic tissue inflammation, and the macrophages’ dysfunction is associated with a variety of diseases including obesity, metabolic syndrome, asthma, allergies, and autoimmune disorders [22]. Thus, M1-like macrophages derived from THP-1 cells were exposed to 10 nM–10 µM DINCH or MINCH with lipopolysaccharides (LPS) in order to simulate the normal activation process, which is the crucial step at the beginning of the innate immune response. Time-dependent responses were examined after 4 h, 16 h, and 24 h of exposure. Cell viability at the concentrations used was verified. Significantly altered proteins of the differentiated and LPS-stimulated THP-1 cells were examined by global proteomics after exposure to the compounds, and enriched signaling pathways and key drivers were analyzed, revealing insights into the mode of action of DINCH and MINCH. Finally, some of these results were validated by examining the release of reactive oxygen species (ROS) and the cytokines TNF and IL-1β.

## 2. Materials and Methods

### 2.1. Cell Culture and Differentiation

Human acute monocytic leukemia THP-1 cells were cultured in growth medium consisting of RPMI 1640 medium (Gibco^TM^, Thermo Fisher Scientific, Waltham, MA, USA) supplemented with 10% fetal bovine serum (FBS, Biowest, Nuaillé, France) and 1% penicillin/streptomycin (Sigma Aldrich, Darmstadt, Germany) at 5% CO_2_, 37 °C, and 95% humidity. Differentiation into adherent M1-like macrophages was induced by incubating 1 × 10^5^ cells per well in a 96-well plate or 2 × 10^6^ cells per well in a 6-well plate in medium supplemented with 100 ng/mL phorbol-12-myristate-13-acetate (PMA, Sigma Aldrich) for 48 h.

### 2.2. Stimulation with Xenobiotics

Cyclohexane-1,2-dicarboxylic acid diisononyl ester (DINCH, CAS no. 166412-78-8, product no. AB440048, 98% purity, ABCR, Karlsruhe, Germany) and monoisononyl cyclohexane-1,2-dicarboxylate (MINCH, product no. C987305, 98% purity, TRC, Toronto, ON, Canada) were commercially purchased and dissolved in methanol (MeOH, MS-grade). DINCH is a mixture of isomers which could induce different effects and can be metabolized into a variety of transformation products [19]. Moreover, the exact amount and ratio of DINCH metabolites resulting from either form are unknown [23]. The manufacturers did not disclose any information on the production and isomer ratio of DINCH and MINCH. Differentiated and adherent THP-1 macrophages were incubated in growth medium containing 10 µM, 1 µM, 0.1 µM, or 0.01 µM DINCH or MINCH in MeOH (0.01%, *v/v* in the medium) and with 0.01 µg/mL lipopolysaccharide (LPS) for 4 h, 16 h, or 24 h. Vehicle controls with or without LPS contained 0.01% (*v/v*) MeOH.

### 2.3. Cell Viability Assay

To check cell viability after stimulation, the CellTiter 96^®^ Aqueous One Solution Cell Proliferation MTS assay (Promega, Walldorf, Germany) was performed according to the manufacturer’s protocol. Briefly, 100 µL of fresh medium and 20 µL of the MTS reaction solution were added to the cells at the end of stimulation and incubated for 1 h at 37 °C. The absorbance was measured at 450 nm in a 96-well Synergy^TM^ HT plate reader (BioTek, Winooski, VT, USA). Treatment with 0.01% (*v/v*) Triton X-100 (Serva Serving Scientists, Heidelberg, Germany) was used as a minimal viability control. The experiment was performed in groups of four.

### 2.4. Endotoxin Assay

To check for endotoxin contamination, DINCH and MINCH were subjected to the Limulus Amebocyte Lysate (LAL) assay using the Pierce^TM^ Chromogenic Endotoxin Quant kit (Thermo Fisher Scientific, Waltham, MA, USA). The assay was performed according to the manufacturer’s protocol using 10 µM DINCH and MINCH dissolved in MeOH.

### 2.5. Measurement of Cytokine Concentrations

TNF and IL-1β concentrations in the conditioned media were determined using the BD OptEIA^TM^ Human IL 1β and BD OptEIA^TM^ Human TNF enzyme-linked immunosorbent assays (ELISA) kit (BD Bioscience, Piscataway, NJ, USA) according to the manufacturer’s protocols. Individual samples were assayed in technical duplicates, and all samples were analyzed in quadruplets. Absorbance was measured at 450 nm and 570 nm. The mean values of 450 nm–570 nm were calculated, and the concentrations were determined using a standard curve.

### 2.6. Quantification of Reactive Oxygen Species (ROS)

ROS in the conditioned medium of THP-1 macrophages were quantified using the DCFDA Cellular ROS Detection Assay Kit (Abcam, Cambridge, UK) according to the manufacturer’s protocol. In brief, in a 96-well plate, the supernatant of differentiated THP-1 macrophages was discarded, and the cells were washed with 100 μL of kit buffer. The cells were then incubated with 100 μL 20 μM 2’,7’-dichlorofluorescein diacetate (DCFDA) staining solution for 45 min at 37 °C in the dark. After another wash step with 100 µL of buffer, cells were treated with 100 µL of 0.01 µM, 0.1 µM, 1 µM, and 10 µM DINCH or MINCH in supplemented buffer (buffer containing 10% FCS, *v/v*). MeOH (0.01%, *v/v*) was used as a negative control and 10 µM tert-butyl hydroperoxide (TBHP, Abcam) as a positive control in supplemented buffer. Fluorescence was measured at Ex/Em 485/525 nm at regular intervals between 5 min–180 min. Experiments were performed in quadruplets and evaluated relative to the MeOH control using mean values.

### 2.7. Statistical Analysis

Besides the proteomics data, all other data are described as means ± standard deviation (SD) and analyzed by one-way ANOVA, including Dunnett’s or Fishers LSD post-hoc tests using the GraphPad 8 software (GraphPad Software Inc., La Jolla, CA, USA).

### 2.8. Proteomics Sample Preparation

After treatment in 6-well plates, THP-1 macrophages were washed three times with 3 mL ice-cold phosphate-buffered saline (PBS, Lonza, Basel, Switzerland) and harvested with lysis buffer containing 150 mM NaCl, 1% Triton X-100, 50 mM Tris HCl pH 7.4, 0.5% sodium deoxycholate, and 0.1% sodium dodecyl sulfate in ddH_2_O supplemented with cOmplete Roche protease inhibitor (Sigma Aldrich). After 1 h incubation on ice, lysates were centrifuged for 15 min at 4 °C and 16,000× *g*. Protein concentration was determined by DC assay (Bio-Rad, Feldkirchen, Germany) according to the manufacturer’s instructions.

Lysates were prepared by single-pot solid-phase-enhanced sample preparation (SP3) based on previous publications in combination with a tandem mass tag (TMT) labeling strategy (TMT-10-plex) for protein quantification [24,25,26,27]. Briefly, 20 µg per sample of SeraMag beads (Sigma Aldrich) were washed with ddH_2_O, incubated on a DynaMag^TM^ magnetic rack (Life Technologies AS, Oslo, Norway) for 2 min, and the supernatant was discarded. The washing step was repeated twice. Beads were transferred to a 96-well plate, washed with ddH_2_O, and the supernatant was discarded after incubation on the magnet rack. Vehicle controls containing 0.01% (*v/v*) MeOH were pooled before being split into four technical replicates during sample preparation. 25 µg of protein from each sample lysate was filled up to a volume of 100 µL with 100 mM tetraethylammonium bromide (TEAB, Sigma Aldrich). Proteins were reduced with 5 µL of 200 mM tris-(2-carboxyethyl)-phosphine (TCEP) hydrochloride (Sigma Aldrich) in 100 mM TEAB buffer for 1 h at 55 °C and then alkylated with 5 µL of 375 mM 2-iodoacetamide (IAA, Merck KGaA, Darmstadt, Germany) in 100 mM TEAB buffer for 30 min at room temperature in the dark. 5 µL of 10% (*v/v*) formic acid (FA, Carl Roth GmbH + Co. KG, Karlsruhe, Germany) and 150 µL of acetonitrile (ACN, Merck KGaA) were added to induce binding of the proteins to the beads in the next step. The samples were transferred to the 96-well plate containing the prepared beads and incubated for 8 min. The supernatant was discarded after a 2 min incubation on the magnetic rack. The beads were washed twice with 70% (*v/v*) ethanol and once with ACN on the magnet rack. After removing the supernatant, the beads were air dried for 15 min. 5 µL of 1 µg/µL trypsin (Promega GmbH, Walldorf, Germany) (protein:trypsin ratio of 1:50) in 100 mM TEAB buffer was added to each well for proteolytic cleavage. 200 μL ddH_2_O was added to the empty wells, and the plate was sealed with parafilm to counteract evaporation during the subsequent 16 h incubation period at 37 °C.

For TMT labeling, 0.8 mg TMT label of TMT-10-plex labeling reagent set (Thermo Fisher Scientific) was dissolved in 42 μL ACN with occasional vortexing for 5 min. 5 μL of TMT label was added to each well and incubated for 1 h at room temperature on the magnet rack. To stop the labeling process, 1 μL of 5% hydroxylamine solution (Thermo Fisher Scientific) in 100 mM TEAB buffer was added and incubated for 15 min. 170 μL ACN was added to achieve an organic content of >95% to promote peptide binding to the beads. Samples belonging to the same 10-plex mix were combined and incubated for 8 min outside the magnet rack. After 2 min on the magnet rack, the supernatant was removed, and the beads were carefully washed with 1 mL ACN.

The first fraction was eluted by adding 200 μL 87% (*v/v*) ACN and ammonium formate (pH 10, Sigma Aldrich) in ddH_2_O, rinsing the solution over the beads five times before transferring to a new tube. For the second fraction, 50 μL ddH_2_O containing 2% DMSO (*v/v*) were added to the beads, sonicated for 1 min, centrifuged, and transferred to a new tube after incubation on the magnetic rack. Again, 50 µL of ddH_2_O containing 2% DMSO (*v/v*) were added, pipetted, incubated on the magnetic rack for 2 min, and the supernatant was combined with the second fraction.

The fractions were evaporated and the pellets were dissolved in 20 µL ddH_2_O by sonication for 5 min. To remove any remaining beads, the solutions were incubated again on the magnet rack for 2 min. The supernatants were transferred to mass spectrometry tubes and acidified with 0.5 µL of 10% (*v/v*) FA.

### 2.9. Proteomics Using LC-MS/MS

The fractionated peptides were separated using an Ultimate 3000 nano ultra-performance liquid chromatography system (Thermo Fisher Scientific) as described before [28]. Samples were first trapped on an Acclaim PepMap 100 C18 column, nanoViper, 2 μm, 75 μm × 5 cm (Thermo Fisher Scientific) and then separated on an analytical reverse-phase column Acclaim PepMap 100 C18, nanoViper, 3 μm, 75 μm × 25 cm (Thermo Fisher Scientific) using a mixture of hydrophilic solution A (0.1% FA in H_2_O, *v/v*) and hydrophobic solution B (80% ACN, 0.1% FA in H_2_O, *v/v*) corresponding to a non-linear gradient of 180 min and a flow rate of 0.3 µL/min. The separated peptides were injected into a Q Exactive HF Hybrid Quadrupole Orbitrap mass spectrometer (Thermo Fisher Scientific) equipped with a TriVersa NanoMate system (Advion, Ihaca, NY USA). The samples were analyzed using MS parameters as previously described, except of selecting top 15 precursor ions of each MS1 scan for fragmentation [28]. MS raw data were processed using ProteomDiscoverer (2.4.0.305, Thermo Fisher Scientific). The database search was performed using the UniprotKB/Swissprot reference proteome of *Homo sapiens* (6 March 2019). The analysis resulted in replicate-wise TMT-reporter ion intensity fold changes (FCs) for 3865 proteins of treatment vs. control without LPS (Supplement 2) or treatment vs. control with LPS (Supplement 3) and were used for subsequent analyses. MS data have been deposited to the ProteomeXchange Consortium via the PRIDE [29] partner repository with the dataset identifier PXD027744 and 10.6019/PXD027744.

### 2.10. Analysis of Proteomics Data

MS data were subjected to statistical analysis with R 3.6.1 using the packages plyr [30], reshape2 [31], xlsx [32], ggsci [33], circlize [34], calibrate [35], ggplot2 [36], readxl [37], qpcR [38], splitstackshape [39], tidyr [40], and Tmisc [41]. Protein FCs were log2 transformed, median normalized and filtered for proteins quantified in at least three of four replicates. The analysis resulted in FCs for 3371 proteins. Significantly altered proteins between treatments and control were calculated using Student’s t-tests (*p* ≤ 0.05; Supplement 4 for treatment vs. control without LPS and Supplement 5 for treatment vs. control with LPS; List of gene names Supplement 6). 

Significantly changed proteins were assigned to pathways using Ingenuity Pathway Analysis software (IPA, QIAGEN Bioinformatics, Hilden, Germany). Parameters such as the species human, immune cells, immune cell lines, and macrophage cancer cell lines as tissue and cell line, as well as a *p*-value cut-off of 0.05, were defined. Pathways were considered significantly enriched with Benjamini and Hochberg adjusted *p*-value ≤ 0.05 (Supplement 7 for treatment vs. control without LPS and Supplement 8 for treatment vs. control with LPS). Given z-scores reflect activation (z-score > 0) or inhibition (z-score < 0) of the pathway. Furthermore, NF-κB target proteins were extracted from the IPA knowledge base. Obtained Entrez Gene IDs were mapped to Uniprot Accessions using the DAVID Bioinformatics Resources 6 [42]. 

Using R 3.6.1, normalized and filtered protein Log2(FCs) were subjected to Weighted Gene Correlation Analysis (WGCNA) [43,44]. A signed network with 10 modules was created (Figure S4) applying the default parameters with following exceptions: soft power threshold: 7, minimum module size: 50, maximum module size: 200, deepsplit: 1, merge cut height: 0.1. Afterwards, modules were correlated to traits using Pearson correlation and Student asymptotic *p*-values. The trait matrices contained DINCH and MINCH treatments, which were distinguished into time points, but combined by their concentrations (Supplement 9). For each of the supposedly interesting module-trait combinations, key drivers were obtained with gene significance and module membership ≥ 0.4 (Supplement 10).

## 3. Results

### 3.1. DINCH and MINCH Have No Cytotoxic Effect in Macrophages at Physiologically Relevant Concentrations

**Figure 1:**
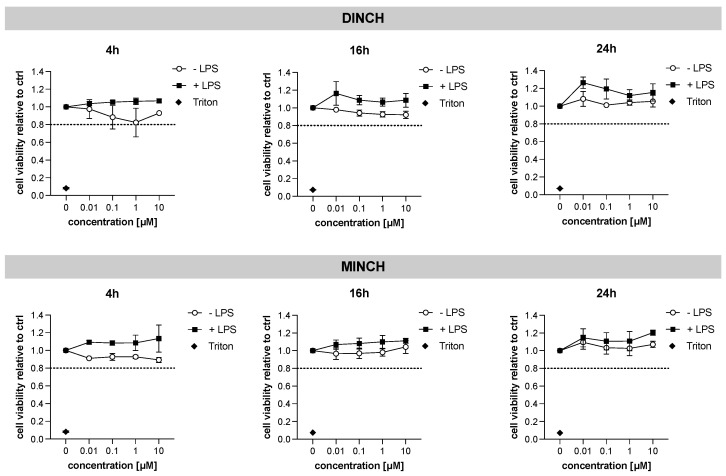
Effects of DINCH and MINCH on the cell viability of THP-1 M1-like macrophages. Differentiated THP-1 cells were treated with increasing concentrations of DINCH or MINCH for 4, 16, and 24 h, followed by MTS-Assay. Treatment with triton (0.01%) served as control reflecting minimal cell viability. Cell viability was determined relative to the control which contained equivalent amounts of vehicle solvent (0.01% MeOH, *v/v*). A decrease in cell viability by 20% (IC_20_) is indicated by a dotted line. Values are displayed as mean ± SD, *n* = 4.

First, to analyze the cytotoxic effects in human macrophages, THP-1 cells were treated with increasing concentrations of DINCH or its metabolite MINCH for 4, 16, and 24 h. To evaluate the effects under acute inflammation, the cells were additionally treated with lipopolysaccharide (LPS). Neither DINCH nor MINCH showed a relevant effect (20% decrease in cell viability: IC_20_) on the viability of THP-1 macrophages at the indicated concentrations or time points (Figure 1).

### 3.2. Proteomic Analysis of the Cellular Effects of DINCH and MINCH Indicate Activation of the NF-κB Signaling Pathway

To unravel the molecular mechanisms induced by DINCH and MINCH, we applied an untargeted LC-MS/MS proteomics approach with tandem mass tag (TMT)-based quantification, which has been shown to be the most appropriate method for proteomics approaches in toxicology [28]. 

**Figure 2:**
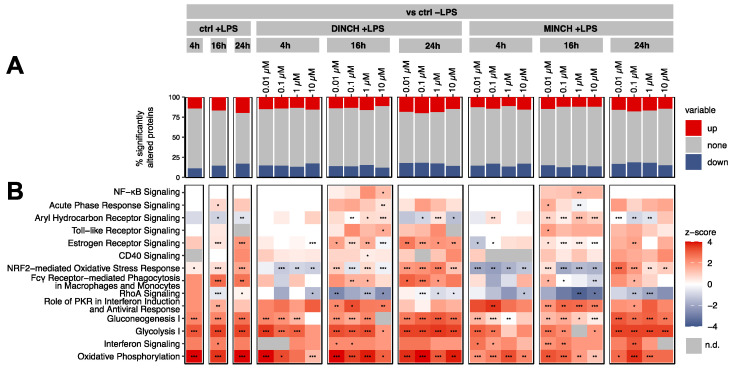
Proteomics analysis of DINCH and MINCH in THP-1 M1-like macrophages using Log2(FCs) of treatment vs. unstimulated control (ctrl –LPS). Differentiated THP-1 cells were treated with increasing concentrations of DINCH and MINCH for 4, 16, and 24 h. Fold changes (FCs) of the treatments to the unstimulated control (–LPS) containing an equivalent amount of MeOH were calculated. (**A**) Percentage of significantly (*p* ≤ 0.05, *n* = 4) altered proteins which were subjected to Ingenuity Pathway Analysis (IPA). (**B**) Heatmap of selected significantly (adjusted *p* ≤ 0.05) enriched pathways in treated THP-1 cells. The heatmap is colored based on z-scores calculated by IPA, indicating the direction of pathway regulation (z-score > 0 pathway is up-regulated, z-score < 0 pathway is down-regulated, not detected is marked in grey). Significant changes are labeled with asterisks (* *p* ≤ 0.05, ** *p* ≤ 0.01, *** *p* ≤ 0.001, *n* = 4).

THP-1 macrophages were stimulated with LPS and simultaneously treated with 0.01 µM–10 µM DINCH or MINCH. To distinguish the effect of DINCH and MINCH from the LPS effect, two controls were used: One containing cells exposed to LPS and equivalent amounts of MeOH (ctrl +LPS), the other containing MeOH but no LPS (ctrl -LPS). For pathways analysis, ratios of DINCH/MINCH treatments and ctrl +LPS were calculated to the ctrl -LPS. A total of 3371 proteins were reliably quantified in three of four replicates. The number of significantly changed proteins (*p* ≤ 0.05) showed that more changes were induced by LPS than by the effect of DINCH or MINCH only (Figure 2A and Figure S3B). Nevertheless, differences in the number of changed proteins were observed between treatments but with no apparent concentration dependence (Figure 2A).

Significantly enriched pathways (adjusted *p* ≤ 0.05) were identified using ingenuity pathway analysis (IPA) based on significantly (*p* ≤ 0.05) altered proteins (Supplement 1, Figure S1). Among the affected pathways, we found immune-related pathways such as NF-κB signaling, Toll-like Receptor signaling, CD40 Signaling, Acute Phase Response Signaling, and Role of PRK in Interferon Induction, which were increased by treatment with DINCH and MINCH at individual time points when compared to the LPS-stimulated control (ctrl + LPS) (Figure 2B) This was also confirmed by the observed changes in specific proteins involved in NF-κB signaling (Supplement 1, Figure S2C). 

The pathway Fcγ Receptor-mediated Phagocytosis in Macrophages and Monocytes showed concentration-dependent regulation after 16 h and 24 h with DINCH and MINCH, respectively, which was less under these conditions compared to the LPS-stimulated vehicle control only (Figure 2B; Supplement 1, Figure S2B). In this context, RhoA Signaling is also noticeable. More prominent than Fcγ Receptor-mediated Phagocytosis in Macrophages and Monocytes, RhoA Signaling was downregulated by DINCH and MINCH, particularly in a concentration-dependent manner for MINCH at 16 h and 24 h (Figure 2B; Supplement 1, Figure S2B).

In addition to immunological signaling pathways, metabolic signaling pathways such as Gluconeogenesis I, Glycolysis I, and Oxidative Phosphorylation also provide information about the status of the immune response. All three pathways were upregulated in the LPS stimulated vehicle controls (ctrl +LPS) as well as by DINCH and MINCH, although upregulation appears to be attenuated by DINCH and MINCH in a concentration-dependent manner (Figure 2B). FCs created to the LPS stimulated control highlight that these signaling pathways were downregulated by DINCH and MINCH treatments (Supplement 1, Figure S2B).

Moreover, LPS upregulated NRF2-mediated Oxidative Stress Response at all three time points. Contrary to that, this pathway was downregulated by MINCH and DINCH at 4 h and 16 h, respectively, until it converged to upregulation at 24 h as in the LPS control (Figure 2B). Furthermore, this trend appeared to be concentration-dependent in MINCH-treated cells.

Notably, the nuclear receptor pathways Aryl Hydrocarbon Receptor Signaling and Estrogen Receptor Signaling were adversely regulated by some treatments with DINCH and MINCH, suggesting a possible interference with signaling of these common xenobiotic-targets [45]. LPS generally upregulated Estrogen Receptor Signaling, but this seemed to be attenuated by DINCH and MINCH at some concentrations after 4 h and enhanced by DINCH after 24 h. This effect is even more evident with FCs created against the LPS stimulated control, showing a downregulation of Estrogen Receptor Signaling for MINCH at all time points and concentrations and a differential regulation for DINCH depending on the time point and concentration (Supplement 1, Figure S2B). Additionally, Aryl Hydrocarbon Receptor Signaling was downregulated by LPS, but upregulated by DINCH and MINCH at 4 h and 16 h.

In summary, several signaling pathways were adversely regulated by DINCH and MINCH. Immune-related signaling pathways were upregulated by DINCH and MINCH, whereas activation of metabolic signaling pathways was decreased (Figure 2B). Furthermore, phagocytosis-associated pathways were downregulated after 16 h and 24 h (Figure 2B). The NRF2-mediated Oxidative Stress Response showed a concentration-dependent and time-dependent pattern, with the initial inhibition of the signaling pathway decaying after 24 h (Figure 2B). Lastly, signaling of two target receptors of endocrine disruption seemed to be impaired.

### 3.3. Keydriver Analysis Reveals Cellular Stress in Response to DINCH and MINCH

**Figure 3:**
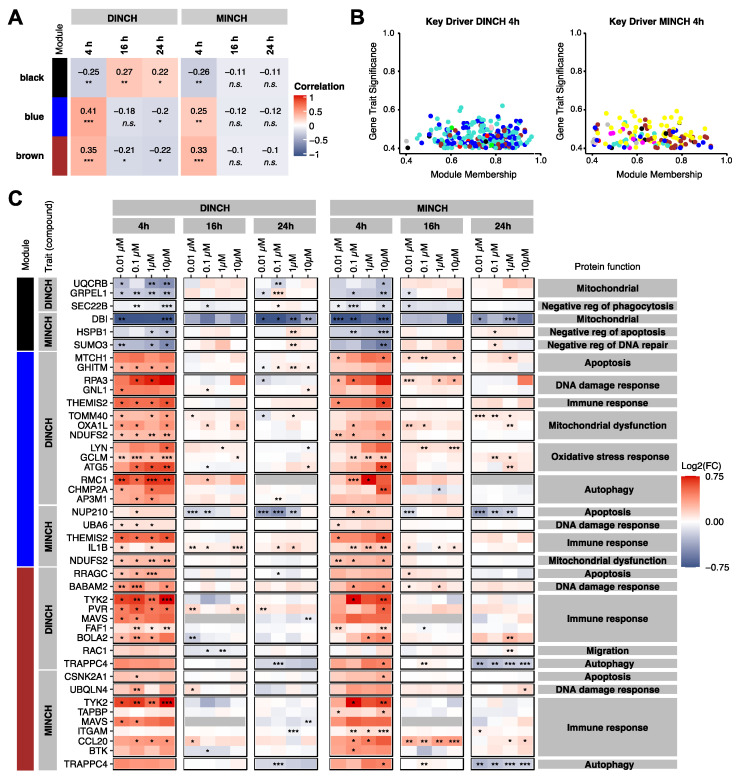
Module-trait correlation and corresponding key drivers obtained by WGCNA. The analysis was based on the Log2(FCs) obtained for the comparison treatment vs. LPS stimulated control (ctrl +LPS). (**A**) Selected modules of co-expressed proteins correlated with compound treatment (DINCH/MINCH) and time point (4 h, 16 h, 24 h). The heatmap is colored according to the correlation, and the significance of the correlation is indicated (n.s. *p* > 0.05, * *p* ≤ 0.05, ** *p* ≤ 0.01, *** *p* ≤ 0.001). (**B**) Identified key drivers (absolute gene significance ≤ 0.4 and absolute module membership ≤ 0.4) for a 4 h exposure to DINCH or MINCH. Proteins are colored based on their assigned module. (**C**) Selected key drivers for modules black, blue, and brown for the trait 4 h. Log2(FCs) are shown relative to the LPS-stimulated control. Significant changes are labeled with asterisks (* *p* ≤ 0.05, ** *p* ≤ 0.01, *** *p* ≤ 0.001, *n* = 4).

To better understand the mode of action of DINCH and MINCH, proteins driving the induced effects were examined using Weighted Gene Correlation Network Analysis (WGCNA) [25,46,47]. For this purpose, Log2(FCs) of DINCH- or MINCH-treated samples vs. the LPS-treated control were used to avoid the LPS effect overlaying the DINCH and MINCH effects. Co-abundant proteins were grouped into 10 color-labeled modules and correlated with traits containing DINCH and MINCH with pooled concentrations but separated time points (Supplement 1, Figure S3). Three modules appeared to be of greater interest because they contained key drivers associated with cellular stress and immune response: blue, brown, and black (Figure 3A), and were considered for further analysis. In general, the 4 h time point showed the most significant correlations with the modules and appeared to correlate in the opposite direction compared to 16 h and 24 h for these modules. According to the correlation values, proteins assigned to the blue and black modules were up-regulated at 4 h and down-regulated at 16 h and 24 h, respectively. The black module showed the opposite direction of regulation. Next, key drivers were identified with focus on those three modules (Figure 3B).

Key drivers of the black module included proteins involved in mitochondrial function, negative regulation of autophagy, DNA repair, and apoptosis (Figure 3C). These proteins were down-regulated after a 4 h exposure to DINCH and MINCH, indicating mitochondrial dysfunction and a promotion of autophagy, DNA repair, as well as apoptosis at this early time point. 

Key drivers of the blue module included proteins involved in apoptosis, DNA damage response, autophagy, and mitochondrial dysfunction. Key drivers of MINCH in the blue module also included proteins involved in the immune response, such as the pro-inflammatory cytokine IL-1β. These proteins also appeared to be up-regulated by DINCH. The key drivers of the brown module were mainly up-regulated and contained proteins related to the immune response, DNA damage response, autophagy, apoptosis, and even migration in the case of DINCH. As shown in the module-trait-matrix (Figure 3A), although the key drivers of the blue and brown modules were generally up-regulated at 4 h, this effect decreased or even reversed at 16 h and 24 h. This pattern was also observed for the black module with down-regulated key drivers at 4 h but an increase of the effect at 16 h and 24 h.

### 3.4. Validation of Proteomics Results Confirms Increased ROS and Cytokine Levels

Proteomics data revealed down-regulation of NRF2-mediated Oxidative Stress Response upon DINCH and MINCH treatments and several key drivers involved in oxidative stress that occurs when ROS production is higher than antioxidant capacity, ultimately leading to cellular damage. Previously, several xenobiotics have been reported to increase ROS formation and trigger an inflammatory response [2] but this has not yet been described for DINCH and MINCH to our knowledge. To investigate whether these compounds induce ROS production, ROS levels were measured after exposure of LPS-stimulated and unstimulated THP-1 macrophages. 

**Figure 4:**
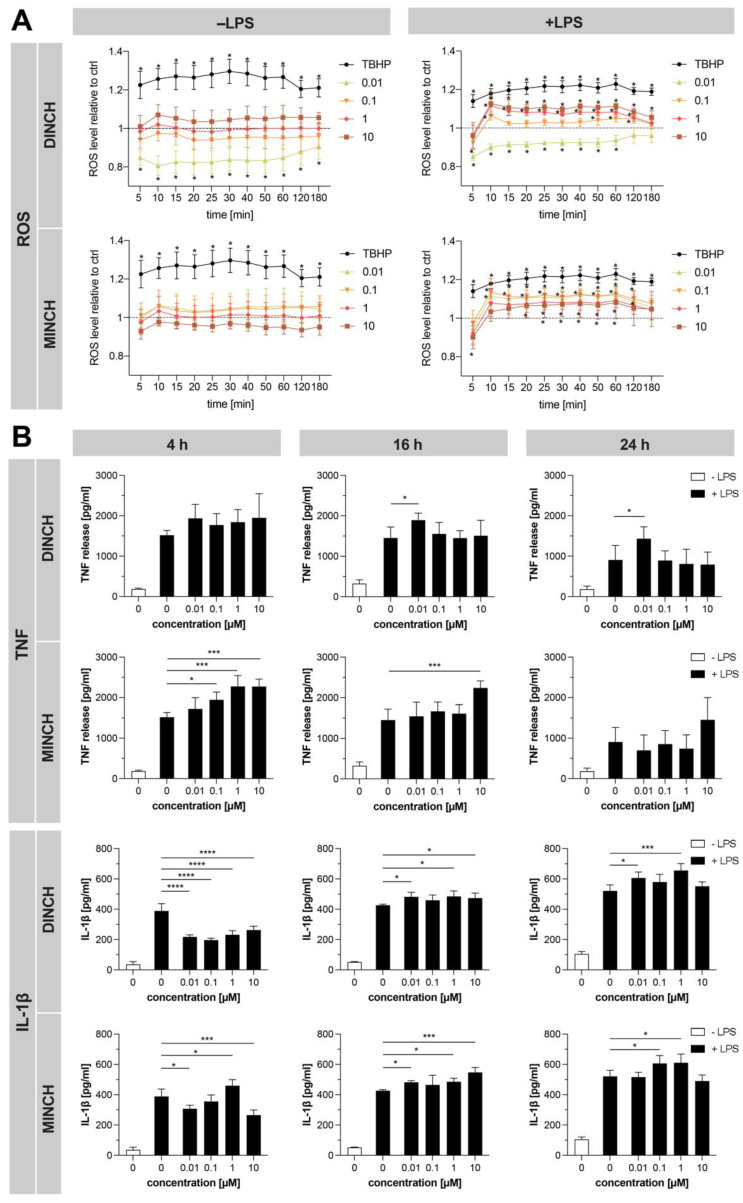
Effect of DINCH and MINCH on intracellular ROS levels and cytokine release. (**A**) ROS levels were measured after THP-1 M1-like macrophage incubation with 20 µM DCFDA for 45 min and treatment with DINCH, MINCH or the positive control TBHP. ROS levels were monitored over 180 min and are shown as ratio to the corresponding control with or without LPS containing equivalent amounts of MeOH Ratios are shown as mean ± SD, *n* = 4. Significant changes are labeled with asterisks (* *p* ≤ 0.05) (**B**) Differentiated THP-1 cells were treated with increasing concentrations of DINCH and MINCH for 4, 16, and 24 h. TNF and IL1-β release was determined in the cell culture supernatant using ELISA and is displayed as mean ± SD, *n* = 4. Significant changes are labeled with asterisks (* *p* ≤ 0.05, *** *p* ≤ 0.001, **** *p* ≤ 0.0001).

For better comparison of the different treatments, the ratio to the control with equivalent amounts of MeOH was displayed. The strong oxidizing agent tert-butyl hydroperoxide (TBHP) served as a positive control and induced the highest increase of ROS levels, which was not matched by DINCH or MINCH (Figure 4A). While increasing DINCH concentrations were accompanied by increased ROS levels, MINCH decreased ROS levels with increasing concentrations (Figure 4A). This pattern was observed with and without concomitant LPS stimulation. Interestingly, ROS levels were not significantly altered without LPS-stimulation, except for 0.01 µM DINCH, which decreased ROS values (Figure 4A). With LPS stimulation, THP-1 cells showed increased ROS levels for all tested concentrations of MINCH and for 1 µM–10 µM of DINCH, while 0.01 µM DINCH again resulted in decreased ROS values (Figure 4A). Overall, ROS levels reached a plateau at 20 min and began to normalize at 120 min (Figure 4A).

In addition, proteomics analysis indicated a pro-inflammatory response as well as significantly increased intracellular IL-1β levels by DINCH and MINCH. Subsequently, release of pro-inflammatory cytokines TNF and IL-1β was validated by ELISA (Figure 4B). We observed a concentration-dependent increase in TNF release in MINCH and LPS-treated THP-1 cells after 4 h, which was sustained at 16 h (Figure 4B). DINCH, on the other hand, increased TNF release at 16 h and 24 h mainly upon treatment with the lowest tested concentration 0.01 µM (Figure 4B). Increased IL-1β release was observed after 16 h and 24 h (Figure 4B). Interestingly, the release was suppressed after 4 h exposure with 0.01 µM–10 µM DINCH as well as 0.01 µM and 10 µM MINCH (Figure 4B). We confirmed that the cytokine release did not occur due to an endotoxin-contamination of DINCH and MINCH stocks using a LAL-assay (Supplement 1, Figure S4).

In summary, higher concentrations (1 µM–10 µM) of DINCH and all tested MINCH concentrations caused a significant increase in ROS levels in LPS-stimulated THP-1 macrophages. While this effect increased in a concentration-dependent manner for DINCH, the opposite was true for MINCH. Both, DINCH and MINCH increased TNF release and IL-1β after 16 h and 24 h.

## 4. Discussion

Since several phthalates have been restricted due to human health concerns, emerging plasticizers also need to be investigated for potential adverse effects as well. One of these emerging alternatives is DINCH, which so far lacks a range of toxicity and endocrine disruptive assessments regarding human health. One of the missing points is the assessment of immunotoxicity, which is crucial for safety evaluation, because immune cells are ubiquitously distributed in various tissues [48] and, in general, plastic additives have been reported to affect inflammatory responses [3,49,50]. Therefore, it is necessary to study the impact of DINCH and its transformation products in immune cells, which we have done here for DINCH and its primary transformation product MINCH in THP-1 M1-like macrophages, since macrophages, as part of the innate immune response, are the first barrier to xenobiotic penetration.

### 4.1. DINCH and MINCH Induce a Pro-Inflammatory Immune Response

The transcription factor NF-κB is a crucial player in pro-inflammatory immune responses. It translocates to the nucleus after degradation of its inhibitor IκB and promotes gene expression of several cytokines, including TNF-α and IL-1β, as well as chemokines, adhesion and co-stimulation molecules [51]. 

Interestingly, proteomics results in our study indicated increased NF-κB signaling for both, DINCH and MINCH. In addition, Toll-like Receptor (TLR) Signaling was upregulated at 16 h and 24 h, indicating TLR-mediated activation of NF-κB. Verifying the observed pro-inflammatory effect and involvement of NF-κB, DINCH and MINCH increased TNF and IL-1β release. Increased blood IL-1β concentrations are associated with the pathogenesis of type 2 diabetes [52], linking DINCH and MINCH to potential adverse health outcomes. 

Underlining this pro-inflammatory response, regulation of metabolic pathways was altered. During inflammation, macrophages increasingly resort to aerobic glycolysis as an energy source, known as the Warburg effect, and for the same reason downregulate the citrate cycle and oxidative phosphorylation to utilize pyruvate for lactate oxidation [53]. Our proteomics analysis showed a concentration-dependent decrease of oxidative phosphorylation by DINCH at 4 h and by MINCH at 16 h compared to the LPS-stimulated control, which was likely mediated by the increased inflammatory response.

A pro-inflammatory effect in the human macrophage cell line THP-1 was previously also described for the commonly used phthalate plasticizer DEHP [12]. DEHP induced translocation of the p65 subunit of the transcription factor NF-κB into the nucleus and, thus, activation of NF-κB, triggering expression of the proinflammatory cytokines such as TNF, IL-1β, IL-6, and IL-8, as well as several chemokines in M1-like macrophages derived from THP-1 cells [10,12], peripheral blood mononuclear cells (PBMCs) [54], and various human [55] and mouse [56] macrophage cell lines. Other phthalate-plasticizers such as di-n-butyl phthalate (DBP) and diisononyl phthalate (DINP) were shown to induce pro-inflammatory cytokines TNF and IL-1β in THP-1 derived M1-like macrophages [21], similar to what we observed for DINCH and MINCH in our study. Additionally, an increase in IL-1β was observed in rats exposed to DBP [57]. For DEHP, Campioli, et al. [58] showed that it caused adipose tissue inflammation, macrophage infiltration, as well as increased serum and adipose tissue TNF levels in male rat offspring after in utero exposure. Moreover, DEHP induced upregulation of immune proteins and genes in zebrafish embryos, including TNF, NF-κB, IL-1β, and IL-8 [59]. Parallels between the pro-inflammatory effects triggered by DINCH in our study and the effects of known endocrine disrupting phthalates in THP-1 macrophages suggest that DINCH may cause comparable adverse effects in vivo. Whether this is indeed the case needs to be clarified in further studies.

The mechanisms underlying the pro-inflammatory effects of DEHP remained unclear and were suggested to partly involve estrogen receptor (ER) signaling [12,54]. Nuclear receptors such as ERs, aryl hydrocarbon receptor (AhR), peroxisome proliferator-activated receptor α (PPARα), and PPARγ are common endocrine disruption targets of plasticizers and can modulate the immune response [45]. In this context, Engel, et al. [60] have shown that while DINCH and MINCH have an inhibitory effect on ERα and ERβ, MINCH can, additionally, activate peroxisome proliferator-activated receptor α (PPARα) and PPARγ. However, the effects were observed at µM concentrations which may not reflect physiological levels, and both compounds showed no impact on AhR activation [60]. Contrary to that, our findings showed an activation of the AhR signaling pathway and indicate an impairment of the estrogen signaling pathway. Estrogen signaling is reported to induce or suppress NF-κB activation, depending on the ligand, and alter cytokine expression [61]. Activation of AhR is known to have an anti-inflammatory effect [62,63]. On the other hand, NF-κB itself induces AhR expression and thus enhances AhR signaling [64]. In accordance with that, the upregulation of the AhR pathway by DINCH and MINCH was mostly driven by NF-κB-associated proteins (data not shown). Consequently, activation of NF-κB by DINCH and MINCH most likely caused the observed increase in AhR signaling. Weather DINCH and MINCH have any impact on ER or AhR activation and directly interact with the ERs requires further investigation.

Notably, the impact on the function of anti-inflammatory M2-like macrophages, which contribute to tissue homeostasis, may differ and is an interesting subject for further studies, as the balance of M1 and M2 macrophages can determine the state of inflammation.

### 4.2. DINCH and MINCH Induce Respiratory Burst and Oxidative Stress in Macrophages

As part of the response to toxins, macrophages can trigger rapid ROS production by enhancing redox reactions and reducing the activity of the antioxidant system [65]. This respiratory burst is essential for pathogen elimination [65]. However, excessive ROS production can lead to oxidative stress and consequent cell and tissue damage [65]. Our results showed that DINCH and MINCH cause macrophages to increase ROS production when stimulated simultaneously with LPS. This effect was concentration-dependent and occurred at high concentrations of DINCH (1 µM and 10 µM) and all concentrations of MINCH. Interestingly, the concentration-response relationship was reversed for MINCH, with the lowest MINCH concentration showing the strongest effect on ROS release. Such a non-monotonic concentration dependence can also be observed for DINCH and MINCH in the proteomic analysis and was a previously described effect of a variety of xenobiotics [66].

The increase in ROS was promoted by a decrease in the abundance and activity of antioxidants, as reflected by the decreased NRF2-mediated oxidative stress response pathway. Finally, ROS levels measured by the DCFDA assay began to slowly decrease again after 2 h of exposure, reflected by the normalization of the NRF2-mediated oxidative stress response at the later 16 h and 24 h time points. This suggests a transient increase in ROS as part of an acute immune response to high DINCH and low MINCH concentrations in stimulated macrophages.

WGCNA analysis of the proteome revealed several key drivers of DINCH and MINCH involved in cellular stress processes, such as DNA damage, mitochondrial dysfunction, and apoptosis. An increase in these key drivers after 4 h indicates that the elevated ROS levels may induce oxidative stress and thereby damage cellular components. 

The reduction of ROS levels to normal levels after 2 h and the decrease in the frequency of key drivers after 16 h indicates recovery of cells from oxidative stress-induced damage. This recovery is also evidenced by the fact that cell viability measured by MTS does not decrease. Nevertheless, under physiological conditions with ubiquitous and continuous exposure to the plasticizer, ROS levels may not decrease after a certain time and cells may fail to recover. The resulting state of chronic oxidative stress can lead to cell death and chronic inflammation, which has been reported to cause and accelerate insulin resistance, pancreatic β-cell death, obesity, and metabolic syndrome [67,68,69]. Induction of oxidative stress was previously also reported for the now restricted plasticizer DEHP and its metabolite MEHP. Both caused mitochondrial dysfunction, increase of ROS generation, and subsequent apoptosis of human lymphoblast cells in vitro [70] and ROS generation and developmental toxicity in zebrafish larvae in vivo [71].

Interestingly, on the one hand, NF-κB activation is reported to regulate the expression or activation of enzymes involved in the production of ROS [72]. On the other hand, it is described that ROS in turn regulate NF-κB activation. This activation can be facilitated by inducing degradation of the NF-κB inhibitor IκB [72]. This interaction links the validated increased ROS levels with the observed upregulation of NF-κB signaling.

### 4.3. Induction of Autophagy for Cell Recovery

Autophagy is a vital cellular process that regulates the lysosomal degradation of aggregated proteins, damaged organelles, and pathogens to maintain cellular homeostasis [73]. In addition, autophagy is associated with apoptosis and may be an adaptation to cellular stress induced by environmental stressors [74]. Xenobiotic-induced apoptosis and associated autophagy have been reported numerous times [75,76,77,78]. Autophagy is thought to play a cytoprotective role in this process to allow cell recovery and survival [74]. Autophagy mechanisms have been described to be induced by cellular stressors such as oxidative stress, DNA damage, and mitochondrial ROS production. By mediating the degradation of proteins and organelles, autophagy can restore cellular homeostasis [74,79]. Damaged or aggregated proteins resulting from oxidative stress are degraded by the autophagy process [74]. Mitochondria damaged by ROS can be removed by autophagy, known as mitophagy, to maintain functional mitochondrial homeostasis in the cell [80]. For DNA damage, a variety of DNA-damage causing agents have been shown to induce autophagy. Moreover, autophagy can be triggered by NF-κB activation or independently of NF-κB by IKK activation of the AMPK and JNK1 pathways [81].

Our proteomic analysis showed that key drivers associated with autophagy processes were upregulated after 4 h, suggesting that treatment of macrophages with DINCH and MINCH induces autophagy. This process could be triggered by increased ROS levels, DNA damage, and mitochondrial dysfunction, which were also observed in the key driver analysis of DINCH and MINCH. This effect subsided after 16 h, reflecting a partial recovery of the cell, suggesting that the macrophages may have been able to degrade the compounds. Nonetheless, the induction of the immune response mediated by DINCH and MINCH appears to be more persistent, as TNF and IL-1β release were still elevated after 16 h and 24 h, respectively. Interestingly, IL-1β is reported to be degraded by autophagy [82], which is reflected in the reduced release of IL-1β at 4 h and the concomitant upregulation of autophagy-associated key drivers at this time point. After 16 h, autophagy processes appear to normalize or even decrease, allowing IL-1β release.

### 4.4. DINCH and MINCH Inhibit Phagocytotic Pathways

In contrast to the upregulation of NF-κB-associated immunoregulatory mechanisms, DINCH and MINCH appear to have an inhibitory effect on phagocytosis, as reflected by the downregulation of the pathways Fcγ receptor-mediated phagocytosis in macrophages and monocytes as well as RhoA signaling for some concentrations. Phagocytosis is one of the most vital functions of pro-inflammatory macrophages, eliminating bacteria and remains of dead cells as well as activating the adaptive immune system by the presentation of the phagocytosed and processed foreign peptide fragments on the surface of macrophages as antigens for B and T cells [1]. RhoA is involved in actin cytoskeleton restructuring and thus phagocytosis [83,84]. Consequently, inhibition of these processes by DINCH and MINCH may negatively affect the inflammatory response to other pathogens and the clearance of an infection. Interestingly, decreased phagocytosis has also been observed with other endocrine disrupting plasticizers, such as DEHP, bisphenol A (BPA) and dibutyl phthalate (DBP) [10].

## 5. Conclusions

**Figure 5:**
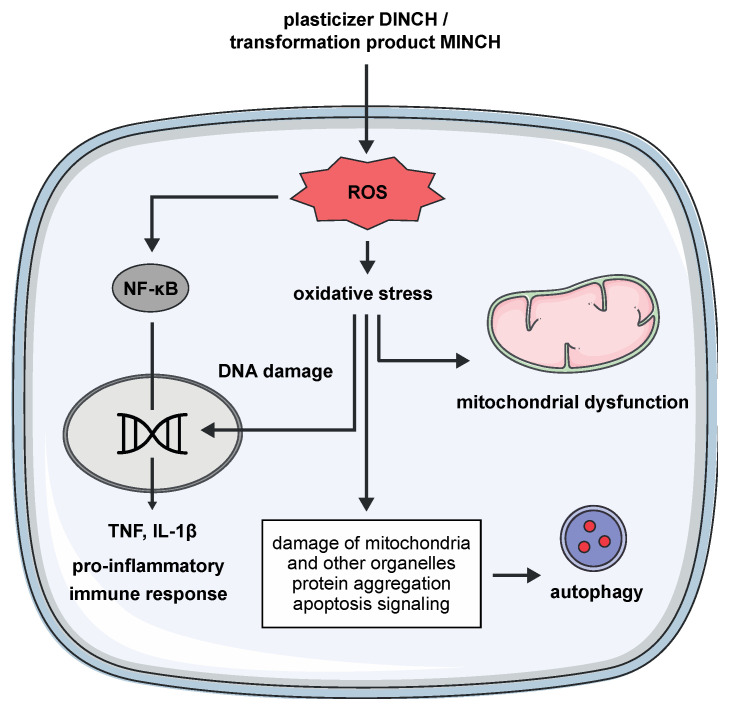
Proposed mode of action of DINCH and MINCH in THP-1 macrophages. ROS levels were increased, leading to activation of NF-κB and subsequent release of TNF and IL1- β, thereby inducing a pro-inflammatory immune response. Increased ROS levels led to oxidative stress, resulting in DNA damage and mitochondrial dysfunction. As a response to the cellular stress and associated apoptosis, autophagy was activated.

We have investigated the effects of DINCH and its primary transformation product MINCH in THP-1 M1-like macrophages, as immunoregulatory effects for these xenobiotics have not been previously studied. We found that both induce a respiratory burst leading to oxidative stress and subsequent DNA damage, mitochondrial dysfunction, and a pro-inflammatory immune response via activation of NF-κB Signaling (Figure 5). In response to the cellular stressors, cytoprotective autophagy processes were triggered, allowing a recovery process (Figure 5). Activation of NF-κB Signaling led to the release of the pro-inflammatory cytokines TNF and IL-1β (Figure 5). Interestingly, these adverse effects were also observed at low nM concentrations, particularly for DINCH. The results described here suggest that the plasticizer DINCH can affect macrophages in humans by inducing cellular stress and thus an inflammatory response. Whether DINCH and MINCH-induced cellular oxidative stress and the resulting inflammatory response may promote inflammation in an exposed organism and might accelerate inflammation-related diseases requires clinical studies.

## Data Availability

Data generated for this manuscript are included in the figures or Supplementary Materials. The proteomics MS data can be found on PRIDE with the dataset identifier PXD027744 and 10.6019/PXD027744.

## Supplementary Materials

The following are available online at https://www.mdpi.com/article/10.3390/cells10092367/s1, Supplement 1: Figure S1: All enriched pathways obtained by Ingenuity Pathway Analysis (IPA) for the exposure of THP-1 macrophages to DINCH and MINCH, Figure S2: Proteomics analysis of DINCH and MINCH in THP-1 macrophages using Log2(FCs) of treatment vs LPS-stimulated control, Figure S3: Full module-trait correlation obtained by WGCNA of treatment vs LPS-stimulated control, Figure S4: Endotoxin-Assay of 10 µM DINCH und MINCH. Supplement 2: FCs of proteins of treatment vs. control without LPS. Supplement 3: FCs of proteins of treatment vs. control with LPS. Supplement 4: Significantly altered proteins for treatment vs. control without LPS. Supplement 5: Significantly altered proteins for treatment vs. control LPS. Supplement 6: List of gene names. Supplement 7: Significantly enriched pathways for treatment vs. control without LPS. Supplement 8: Significantly enriched pathways for treatment vs. control with LPS. Supplement 9: WGCNA trait matrices. Supplement 10: Key drivers were obtained with gene significance and module membership ≥ 0.4.
