# Supplementary material for: The Emerging Plasticizer Alternative DINCH and Its Metabolite MINCH Induce Oxidative Stress and Enhance Inflammatory Responses in Human THP-1 Macrophages
Source: Cells. 2021 Sep 9;10(9):2367. doi: 10.3390/cells10092367 (PMC8466537; doi:10.3390/cells10092367)
Supplement: Supplementary file 1 [file cells-10-02367-s001.zip › Supplement 1.pdf]

Supplement 1

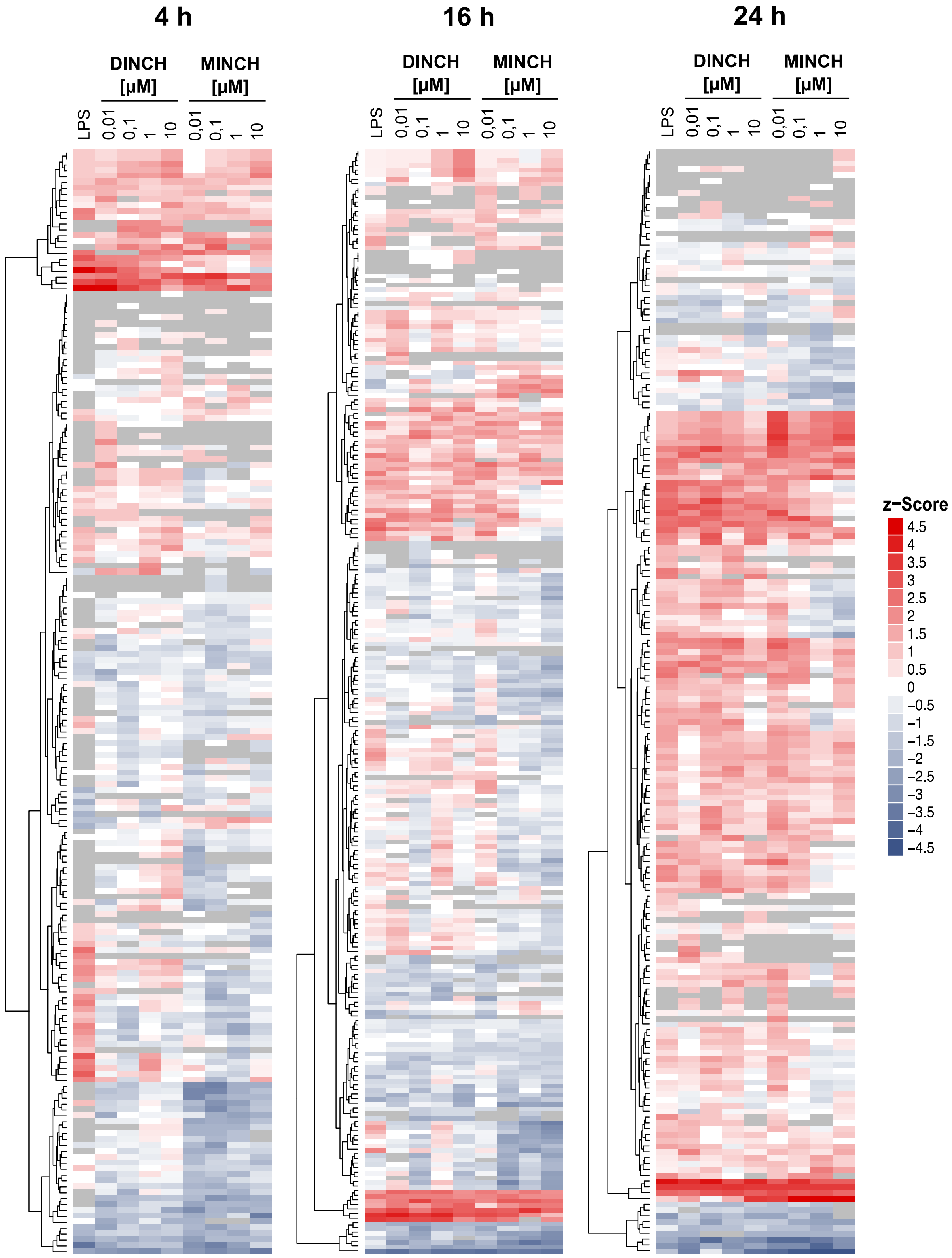

Figure S1: All enriched pathways obtained by Ingenuity Pathway Analysis (IPA) for the exposure of THP-1 macrophages to DINCH and MINCH. *THP-1 macrophages were exposed to 10 nM – 10 µM of compound for 4 h, 16 h, and 24 h. Fold changes were calculated relative to the unstimulated control containing equivalent amounts of solvent (0.01% MeOH). Significant changes are labeled with asterisks (* $p \leq 0.05$, ** $p \leq 0.01$, *** $p \leq 0.001$, n=4).*

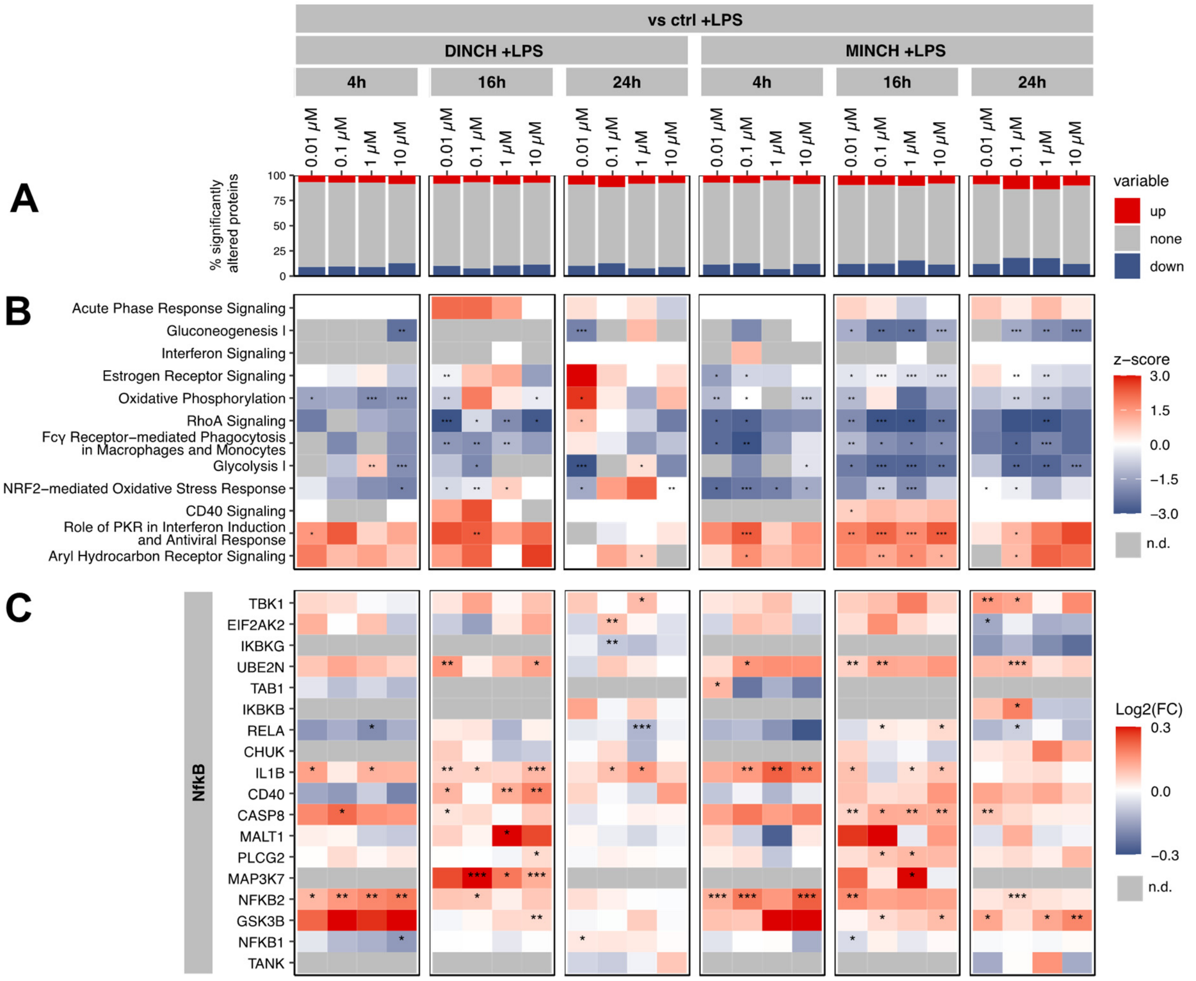

Figure S2: Proteomics analysis of DINCH and MINCH in THP-1 macrophages using Log2(FCs) of treatment vs LPS-stimulated control. *Differentiated THP-1 cells were treated with increasing concentrations of DINCH and MINCH for 4, 16, and 24 h. Fold changes (FCs) of the treatments to the LPS-stimulated control containing an equivalent amount of MeOH were calculated. (A) Percentage of significantly ($p \leq 0.05$, n=4) altered proteins after exposure of THP-1 cells which were subjected to Ingenuity Pathway Analysis (IPA). (B) Heatmap of selected significantly (adjusted $p \leq 0.05$) enriched pathways in treated THP-1 cells. The heatmap is colored based on z-scores calculated by IPA, indicating the direction of pathway regulation (z-score > 0 pathway is up-regulated, z-score < 0 pathway is down-regulated, not detected is marked in grey). Significant changes are labeled with asterisks (* $p \leq 0.05$, ** $p \leq 0.01$, *** $p \leq 0.001$, n=4). (C) NF-κB associated proteins for the exposure of THP-1 macrophages to DINCH and MINCH. Significant changes are labeled with asterisks (* $p \leq 0.05$, ** $p \leq 0.01$, *** $p \leq 0.001$, n=4).*

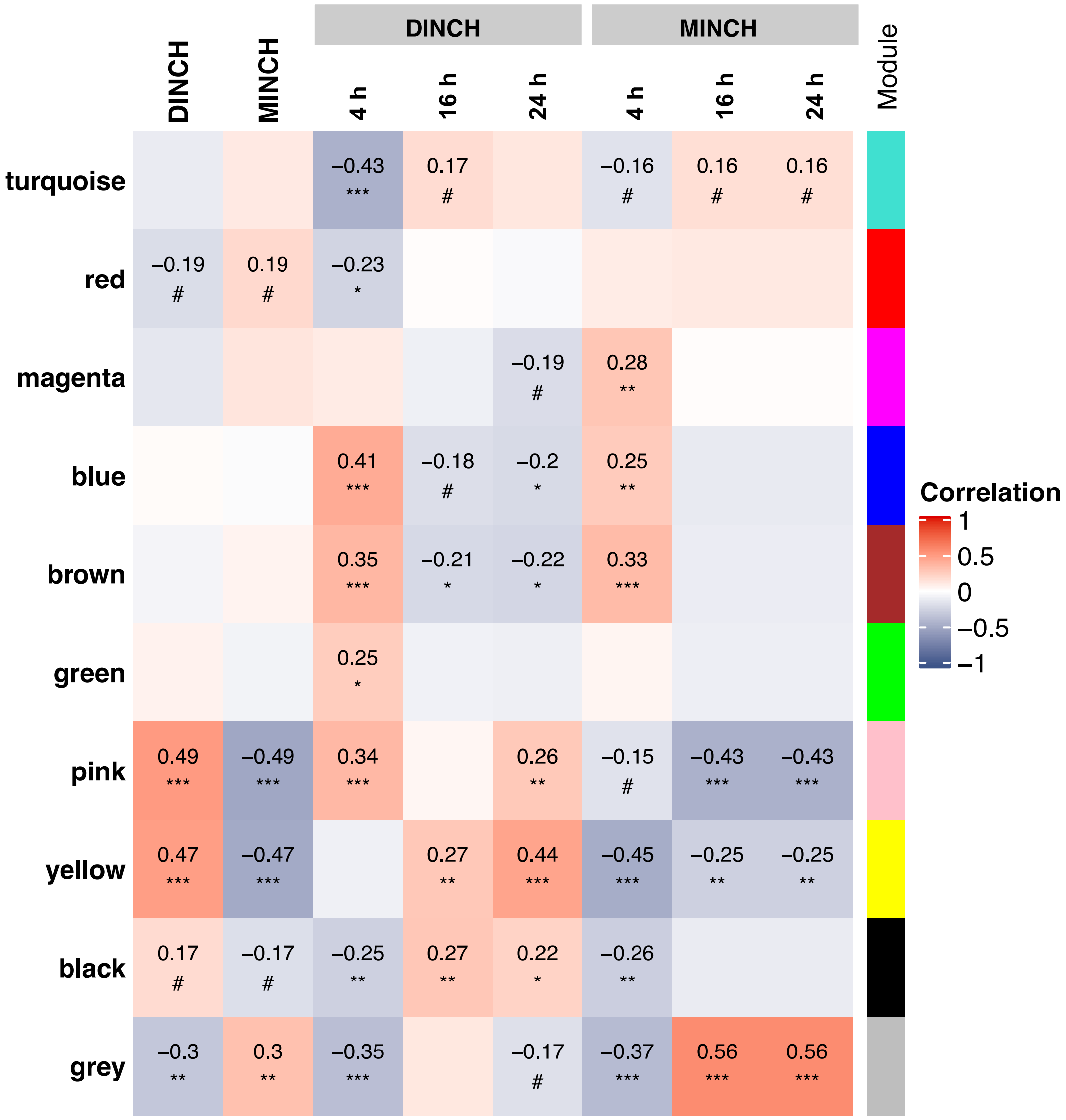

*Figure S3: **Full module-trait correlation obtained by WGCNA of treatment vs LPS-stimulated control.** Displayed are all ten obtained modules of co-expressed proteins correlated with compound treatment (DINCH/MINCH) and time point (4 h, 16 h, 24 h). The heatmap is colored according to the correlation and significance of correlation is indicated (# p ≤0.01, * p ≤ 0.05, ** p ≤ 0.01, *** p ≤ 0.001).*

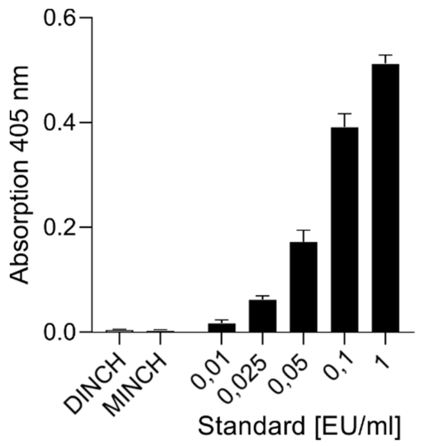

Figure S4: Endotoxin-Assay of 10 µM DINCH und MINCH. *Displayed are the absorbances of the highest tested concentration (10 µM) of DINCH and MINCH and endotoxin standard dilutions. Data are depicted as mean ± SD, n=3.*
